# Enhancing Equity in Schoolchildren’s Basic Life Support Education in Brazil Through Serious Games: Cohort Study

**DOI:** 10.2196/69252

**Published:** 2025-11-10

**Authors:** Uri Adrian Prync Flato, Adriana Flato, Isabella Bispo Diaz T Martins, Giuliana Simoes Nakano, Julia Caroline Romao, Manuela Simões Nakano, Emilio José Beffa dos Santos, Yuuki Daniel Tahara Villas Boas, Leonardo Escobar Medeiros, Vinicius Gazin Rossignoli, Gabriel Zanatta Cunha, Rafael Carreira Batista, Pedro Gazotto Rodrigues da Silva, Miguel Florentino, Amanda Rabelo, Thais Dias Midega, Rogerio Passos, Helio Guimaraes, Karl Kern

**Affiliations:** 1 Critical Care Unit, Hospital Vila Nova Star-Rede D'Or São Paulo/ São Luiz Itaim-Rede D'Or, Dr. Alceu de Campos Rodrigues, 126, São Paulo, 04544-000, Brazil, 55 11975858889, 55 975858889; 2 Sanare Institute, São Paulo, Brazil; 3 Universidade de Marília, Marília, Brazil; 4 Hospital Israelita Albert Einstein, Jose antonio coelho, 626, São Paulo, 04011061, Brazil; 5 Sarver Heart Center, Tucson, AZ, United States

**Keywords:** cardiopulmonary, resuscitation, training, schoolchildren, gamification, basic life support

## Abstract

**Background:**

Out-of-hospital cardiac arrests (OHCAs) predominantly occur in residential settings, often witnessed by children who could act as first responders. The World Health Organization (WHO) supports the Kids Save Lives (KSL) initiative, recommending basic life support (BLS) training for children aged ≥11 years. However, disparities in BLS education persist globally, particularly in low-resource regions where socioeconomic barriers, such as school type, malnutrition, and limited infrastructure, hinder implementation. Younger children (aged <11 years) face additional challenges due to physical limitations (eg, height, weight, and grip strength), which may compromise their ability to achieve adequate chest compression depth. While gamified learning has shown promise in improving BLS engagement and skill acquisition, its efficacy across diverse socioeconomic groups remains understudied.

**Objective:**

This study aimed to compare cardiopulmonary resuscitation (CPR) performance (compression depth, rate, and recoil) between public and private school students following a game-based BLS intervention and evaluate the feasibility of a serious game (Kids Save Hearts) in improving BLS knowledge and irrespective of socioeconomic background.

**Methods:**

We conducted an observational cohort study with 336 students aged 7‐17 years from 10 public and 10 private schools in Brazil (April to November 2022). Participants received 40 minutes of video-based CPR training (American Heart Association CPR in Schools) followed by 10 minutes of gamified training using the Children Save Hearts serious game (SG). CPR quality was assessed via quality of cardiopulmonary resuscitation (QCPR) scores (Laerdal QCPR manikin), measuring compression depth (mm), compression rate (per minute), and chest recoil. Anthropometric data (height, weight, and grip strength) and socioeconomic indicators (school type) were collected. Nonparametric tests (Mann-Whitney *U* and chi-square tests) and multivariate regression (SPSS v27.0; IBM Corp) were used to analyze associations between demographics, physical characteristics, and CPR performance.

**Results:**

Older students (11‐17 years) outperformed younger peers (7‐10 years) in median compression depth (48 mm vs 37 mm; *P*<.001) and overall QCPR scores (84 vs 42, *P*<.001). Private school students had higher grip strength (24.92 vs 21.48 g/cm²; *P*=.001), but school type did not significantly affect CPR quality. Postintervention SG scores improved universally (*P*<.001), with no age or socioeconomic disparities. Multivariate analysis identified age (*P*<.001), height (*P*<.001), and grip strength (*P*<.001) as independent predictors of high QCPR scores (≥70).

**Conclusions:**

Age and physical development were stronger determinants of CPR quality than socioeconomic factors. The game-based intervention effectively improved BLS knowledge and skills across all participants, demonstrating its potential as an equitable training tool. These findings support the scalability of gamified BLS programs in resource-limited settings.

## Introduction

### Background

Cardiovascular diseases remain the leading cause of mortality worldwide, accounting for 80% of deaths [1]. Out-of-hospital cardiac arrests (OHCAs) frequently occur at home [2], often in the presence of family members, including children. Over the past 2 decades, OHCA survival rates have improved in North America, Europe, and Asia [3-5], attributed to early recognition, prompt emergency system activation, high-quality chest compressions, and rapid defibrillation. However, achieving similar outcomes globally requires transformative public policies, such as those recommended by the Institute of Medicine in 2015 [6]. Strengthening the chain of survival hinges on layperson training in basic life support (BLS), particularly in high-risk populations [7].

The World Health Organization (WHO) endorses the Kids Save Lives (KSL) initiative [8], advising that children aged 11 years and older receive at least 2 hours of annual BLS and automated external defibrillator training [9]. However, the WHO does not prescribe specific methodologies, leaving room for innovation, particularly through digital tools. Disparities in access to BLS education further complicate implementation, with students in public schools often lacking resources (eg, manikins and trained instructors) compared to their private school counterparts. Socioeconomic factors, including malnutrition and delayed physical development, may also impair cardiopulmonary resuscitation (CPR) performance in underserved communities. Addressing these inequities demands adaptable solutions, such as low-cost manikins, strength-building exercises, and inclusive, technology-driven teaching strategies.

Schoolchildren are ideal first responders and multipliers of BLS knowledge within their communities. Evidence suggests that gamified learning can enhance engagement and long-term retention of CPR skills in children [10]. Serious games, combined with quantitative psychomotor assessments, offer a promising avenue for scalable BLS training [11]. Yet, few studies have evaluated the impact of such interventions across socioeconomic strata.

### Study Objective

This study aimed to evaluate disparities in CPR quality (chest compression depth, rate, and chest recoil) between public and private school students following a game-based BLS training intervention (Kids Save Hearts Project), assessing the feasibility of digital tools to bridge gaps in life-saving competency.

## Methods

### Overview

**Figure 1.**
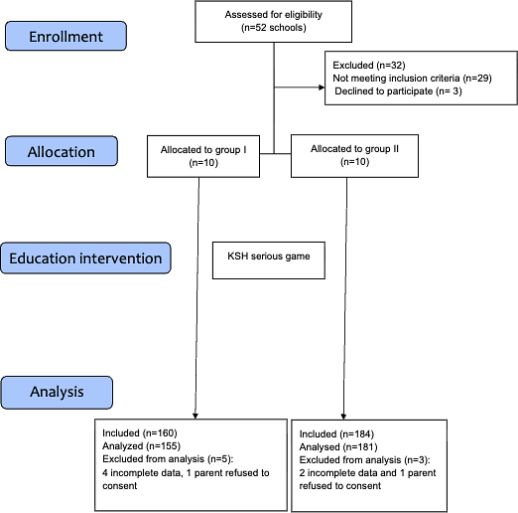
Study design flowchart. KSH: Kids Save Hearts.

This study was designed as an observational cohort study following the STROBE (Strengthening the Reporting of Observational Studies in Epidemiology) [12] guidelines (Figure 1). A total of 328 children from elementary and high schools were included, divided into 2 groups based on age: 7-10 years (Group I) and 11-17 years (Group II). Schools, both public and private, were selected through a scientific cooperation agreement with the University of Marilia, located in the western region of São Paulo, Brazil. Selection was conducted sequentially, alternating 1:1 between public and private schools. Classes were enrolled to match the required sample size, considering an average of 20 students per class. The study took place from April to November 2022. Schoolchildren and their legal guardians provided written informed assent and consent to participate. Inclusion criteria required participants to be enrolled in elementary or secondary school, have no prior training in BLS, and provide informed assent and consent. Exclusion criteria included previous CPR training or inability to attend all planned activities (Textbox 1). CPR training was not previously part of the Brazilian educational curriculum in elementary and high schools.

Textbox 1. **Inclusion and exclusion criteria for participant selection in the cohort study.**

**Inclusion criteria**

- Participants enrolled in elementary or secondary school
- No prior training in basic life support (BLS)
- Provision of informed assent and consent

**Exclusion criteria**

- Previous cardiopulmonary resuscitation (CPR) training
- Inability to attend all planned sessions

### CPR Training

**Figure 2.**
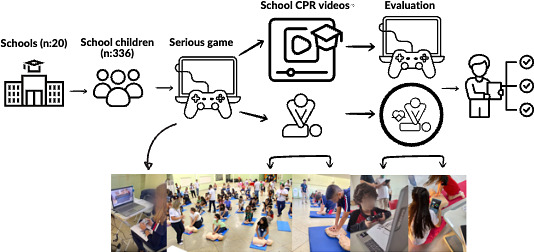
Cardiopulmonary resuscitation training flow: the implementation framework traditional video-based cardiopulmonary resuscitation instruction with supplemental digital game-based learning (KSH serious game) components, with outcomes measured using Laerdal QCPR (quality cardiopulmonary resuscitation) manikins.

The training was developed following a pattern (Figure 2) that included: welcoming the children, obtaining demographic data (weight, height, bicep circumference, and palmar strength test), qualitative evaluation of the student’s expectations in a written way, serious game (SG) play, video-based training, serious game evaluation (SGT1), postcourse written qualitative perception and evaluation for 1 minute with quality of cardiopulmonary resuscitation (QCPR) manikin. All participants received 40 minutes of video-based training on CPR, practicing while watching based on the American Heart Association (AHA) CPR in Schools Training Kit and 10 minutes of training with continuous chest compressions with audio feedback. The training sessions were conducted with classes of 20 participants during the school period. Mini Little Anne Manikin (Laerdal) and Little Anne QCPR (Laerdal Medical Inc) with real-time feedback software (QCPR training 4.13.3, Laerdal Medical Inc) were used for training. Certified basic life support instructors (AHA) conducted the training based on 2020 resuscitation guidelines [13] with a ratio of 10:1 schoolchild to instructors.

### QCPR Score

The QCPR score is a standardized metric designed to assess the effectiveness of CPR performance by evaluating key parameters, such as chest compression depth, rate, recoil, and ventilation volume. The QCPR score is calculated as a percentage, with higher scores indicating closer adherence to guideline-recommended CPR standards. By quantifying performance, the QCPR score helps identify areas for improvement, ensuring that high-quality CPR is delivered during cardiac arrest scenarios, which is critical for improving patient outcomes.

Evaluation criteria for QCPR focus on the consistency and accuracy of chest compressions and ventilations. Key metrics include maintaining a compression depth of 5‐6 cm, a rate of 100‐120 compressions per minute, complete chest recoil between compressions. Deviations from these targets result in lower QCPR scores, reflecting suboptimal CPR quality. In addition, hands-off time (interruptions in compressions) is factored into the evaluation, as excessive pauses can diminish perfusion efficacy. The QCPR assessment not only serves as a training tool for health care providers but also supports research initiatives aimed at correlating CPR quality with clinical outcomes, reinforcing the importance of adherence to evidence-based resuscitation protocols.

### Serious Game Children Save Hearts

The serious game Children Save Hearts was developed on the Smile Game Builder platform (open source) and implemented on Windows 7, 8, 10, and 11 operating systems. The game was developed with the objective of teaching children from 7 to 11 years old to perform BLS care. The script and storytelling are based on the ILCOR (International Liaison Committee on Resuscitation) guidelines.

In the game, the user must follow commands to perform CPR in an avatar. At the beginning of the game, the user must answer questions on how to conduct the service. At the end, the user receives a grade based on the overall performance. The game includes the use of a joystick and simple commands to make the user experience simpler, allowing greater focus on the content. We define a first contact (time) with the SG as (SGTO) and the evaluation phase of SGT1. A usability evaluation was validated and published previously [14], contemplating 5 elements (recognition of unconsciousness, call for help, initiate fast and strong chest compressions, maintain compressions at least 100 to 120 per minute, and automated external defibrillator) with maximum and minimum game score from 0 to 500.

### Sample Size

The sample size calculation determined that a minimum of 296 participants was required to detect a 12-mm [15,16] difference in chest compression depth with an expected SD of 10 mm, assuming a significance level of 0.05, power of 90%, and an estimated 20% dropout rate. To account for potential exclusions and ensure the representativeness and robustness of the results, we included a total of 336 participants in the study. This approach minimized the risk of underpowering the study due to unanticipated data losses or incomplete participation.

### Statistical Analysis

All statistical analyses were performed using IBM SPSS Statistics (v27.0) with a significance threshold of *P*<.05. Data normality was assessed using the Shapiro-Wilk test, which guided the choice of parametric or nonparametric tests. Continuous variables (compression rate, depth, and QCPR scores) were compared between groups using independent 1-tailed *t* tests (for normally distributed data) and Mann-Whitney *U* tests (for nonnormally distributed data). Categorical variables (school type and gender) were analyzed via chi-square and Fisher exact tests. For correlational analyses, Spearman rank correlation (nonparametric) and Pearson r (parametric) tested associations between anthropometric measures (height, weight, and grip strength) and CPR quality (QCPR scores). Multivariate logistic regression identified predictors of high-quality CPR (QCPR ≥70), adjusting for age, school type, and gender. Pre- and posttraining knowledge gains (serious game scores) were evaluated using Wilcoxon signed-rank tests, while between-group differences (age and school type) were assessed via Kruskal-Wallis tests.

### Ethical Considerations

The study protocol was approved by the Ethics Committee of the University of Marília under registration number CAAE: 57160121400005496 [17] and NCT06565767 [18]. Informed consent and informed assent were obtained from all participants before their inclusion in the study. All collected data were deidentified to ensure participant confidentiality. Participants were not compensated for their involvement, and no identifiable personal information is included in this manuscript. Identifiable images published herein were included with written consent.

## Results

### Study Participants

The study ultimately included 336 participants from 10 public and 10 private schools (Table 1). Among them, Group 1 comprised 155 participants with a median age of 9.2 years, while Group 2 had 181 participants with a median age of 14.4 years. Anthropometric measurements varied among participants, with a notable prevalence of obesity and overweight.

**Table 1.** Baseline characteristics and cardiopulmonary resuscitation (CPR) performance metrics in schoolchildren stratified by age group: a cross-sectional study of 336 participants (Group 1: 7‐10 years, n=155; Group 2: 11‐17 years, n=181).

Variable	Group 1 7‐10 years (n=155)	Group 2 11‐17 years (n=181)	*P* value
Age (years), median (IQR)	9.20 (7‐10)	14.40 (11‐17)	<.001^a^
Male sex, n (%)	78 (50.3)	81 (44.8)	.30^b^
Private school, n (%)	53 (34.2)	51 (28.2)	.28^b^
Height (cm), median (IQR)	141.00 (135.50‐146)	164 (155‐171)	<.001^a^
Weight (kg), median (IQR)	37.60 (30.90-45.75)	60.40 (49.80‐69.90)	<.001^a^
BMI			
Underweight, n (%)	6 (4)	10 (5)	.24^b^
Normal weight, n (%)	79 (51)	119 (65)	<.001^b^
Overweight, n (%)	37 (24)	30 (16)	<.001^b^
Obese, n (%)	33 (21)	22 (12)	<.001^b^
Biceps circumference (cm), median (IQR)	23 (20.50-25.50)	27 (24‐30)	<.001^a^
HGS^c^ (g/cm^2^), median (IQR)	18.80 (15-28.50)	24.50 (20.25‐31.70)	<.001^a^
Compression (per minute), median (IQR)	110 (104-123)	107 (104-116)	.01^a^
Chest recoil (%), median (IQR)	98 (96-100)	95 (91-100)	<.001^a^
Compression depth (mm), median (IQR)	37 (31-44)	48 (39-53)	<.001^a^
QCPR^d^ score, median (IQR)	42 (30-64)	84 (55-98)	<.001^a^
Serious game KSH^e^			
T0 Score	350 (350-450)	300 (300-450)	.34^a^
T1 Score	450 (400-500)	450 (400-500)	.29^a^

a Group comparisons used Mann-Whitney *U* tests.

b Chi-square tests.

c HGS: handgrip strength.

d QCPR: Quality of Cardiopulmonary Resuscitation.

e KSH: Kids Save Hearts.

### Chest Compression Performance

#### Compression Rate

There were no significant differences in compression rates between the age groups, with Group 1 performing at a median of 110 compressions per minute compared to Group 2 at 117 compressions per minute (*P*=.01).

#### Compression Depth

Group 2 demonstrated a significantly greater compression depth compared to Group 1, with a median depth of 48 mm in Group 2 versus 37 mm in Group 1 (*P*<.001).

#### Quality of Chest Compressions

The overall quality of chest compressions, as measured by the QCPR score, was significantly better in Group 2, with a median score of 84 compared to 42 in Group 1 (*P*<.001).

### Knowledge Acquisition

Serious game scores: there was a statistically significant improvement in knowledge acquisition posttraining, with serious game scores showing a marked increase (*P*<.001). However, there were no significant differences in posttraining scores between the age groups.

### Comparisons by School Type and Gender

School type (public vs private): the performance of students from public and private schools (Table 2) did not differ significantly overall. However, private school students exhibited a higher median hand grip strength compared to their public-school counterparts (24.92 vs 21.48 g/cm²; *P*=.001). Gender differences: gender did not have a significant impact on CPR performance (Tables1  2), as there were no notable differences based on gender in the results.

**Table 2.** Comparison between the private versus public schools. Analysis of 336 participants (Public School: n=232; Private school: n=104) of urban schools in Marilia, Brazil. Data include sex distribution, age, height, weight, biceps circumference, handgrip strength (HGS), and CPR^a^ quality measures (compression rate, depth, recoil percentage, and QCPR^b^ scores) assessed using Laerdal LittleAnne QCPR manikins. Continuous variables are reported as median [interquartile range (IQR)]; categorical variables as n (%). Group comparisons used Mann-Whitney *U* tests.

Variable	Public school (n=232)	Private school (n=104)	*P* value
Male sex, n (%)	110 (47.4)	49 (47.1)	>.99^c^
Age (years), median (IQR)	11 (10‐16)	10 (10‐14.25)	.29^d^
Height (cm), median (IQR)	153 (141‐165)	150 [142.75‐162.25]	.50^d^
Weight (kg), median (IQR)	49.20 (36.45‐62.02)	48.75 (38.90‐59.95)	.99^d^
Biceps circumference (cm), median (IQR)	25 (22‐28)	25.50 (22‐28.50)	.35^d^
HGS^e^ (g/cm^2^), median (IQR)	21.48 (16‐29.52)	24.92 (20.25‐32)	.001^d^
Compression (minute), median (IQR)	108 (104‐117)	111 (104‐122)	.19^d^
Chest recoil (%), median (IQR)	100 (99‐100)	100 (99‐100)	.39^d^
Compression depth (mm), median (IQR)	44 (35‐50.25)	40 (33.75‐49.25)	.09^d^
QCPR score, median (IQR)	67.50 (40‐91.25)	47 (34.75‐90)	.05^d^

a CPR: cardiopulmonary resuscitation.

b QCPR: quality of cardiopulmonary resuscitation.

c For proportions.

d For continuous variables and chi-square tests*.*

e HGS: handgrip strength.

### Factors Correlated With High-Quality CPR

QCPR score ≥70: factors associated with achieving a QCPR score of 70 or higher (Table 3), indicative of high-quality CPR, included higher age, greater height, weight, biceps circumference, and hand grip strength, with all factors showing significant associations (*P*<.01).

**Table 3.** Characteristics and CPR^a^ performance metrics stratified by QCPR^b^ score (QCPR <70: n=186; QCPR ≥70: n=150) The table compares demographics (sex, school type), anthropometric measures (age, height, weight, and biceps circumference), handgrip strength (HGS), and CPR quality indicators (compression rate, depth, and chest recoil) between participants achieving adequate (QCPR ≥70) versus inadequate (QCPR <70) CPR performance scores. Continuous variables presented as median (IQR); categorical variables as n (%). Statistical comparisons performed using Mann-Whitney *U* test.

Variable	QCPR score <70, (n=186)	QCPR ≥70, (n=150)	*P* value
Male sex, n (%)	87 (46.8)	72 (48)	.90^c^
Private school, n (%)	64 (34.4)	40 (26.7)	.15^c^
Age (years), median (IQR)	10 (9‐11)	15 (11‐16)	<.001^d^
Height (cm), median (IQR)	144 (137‐152.75)	162 (154‐170.75)	<.001^d^
Weight (kg), median (IQR)	40.30 (31.85‐50.27)	60.65 (48.32‐71.22)	<.001^d^
Biceps circumference (cm), median (IQR)	23 (21‐26)	27.75 (25‐30)	<.001^d^
HGS^e^ (g/cm^2^), median (IQR)	20.20 (15.4-26.9)	25.32 (20.3-33.9)	<.001^d^
Compression (per minute), median (IQR)	109 (104‐122.7)	108 (104‐116)	.17^d^
Chest recoil (%), median (IQR)	100 (100-100)	100 (94-100)	<.001^d^
Compression depth (mm), median (IQR)	36 (31-40)	50 (47-55)	<.001^d^

a CPR: cardiopulmonary resuscitation.

b QCPR: Quality of Cardiopulmonary Resuscitation.

c For proportions, with significant differences (*P*<.05) highlighted for key performance metrics including compression depth (*P*<.001) and HGS (*P*<.001).

d For continuous variables and chi-square test.

e HGS: handgrip strength.

### Pre- and Posttraining Knowledge Scores

**Figure 3.**
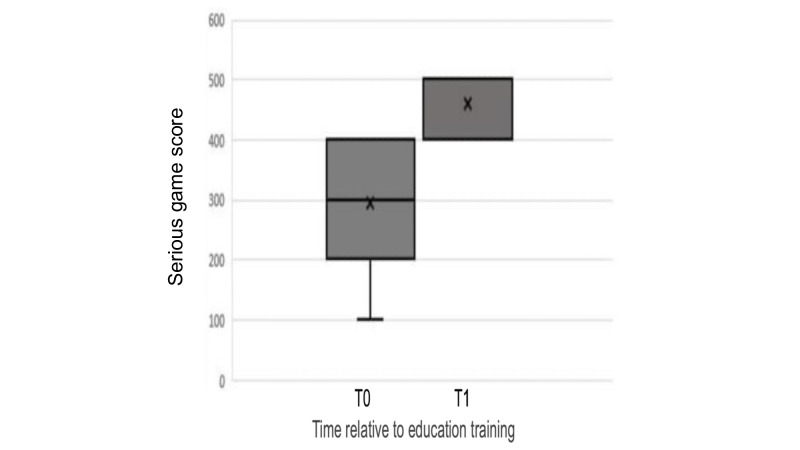
Box plot analysis of CPR knowledge in a kids save hearts serious game: comparative distributions of serious game score before (T0) and after BLS training (T1). CPR: cardiopulmonary resuscitation.

Serious game scores: scores from the serious game (Figure 3) showed a significant increase from pretraining (SGT0) to posttraining (SGT1) for all participants, indicating improved knowledge (*P*<.001).

## Discussion

### Principal Findings

The importance of BLS training in schools has been increasingly recognized [19]. Our study underscores the importance of early and effective CPR training and adds valuable insights into how age and physical attributes influence the quality of chest compressions among schoolchildren.

### Age-Related Performance Differences

Effective bystander CPR is crucial for improving survival rates in cases of OHCA. Research suggests that introducing CPR training to elementary children, starting as early as 4 years old, can significantly impact bystander intervention rates and survival outcomes [16]. Studies indicate that schoolchildren can effectively learn and replicate CPR techniques, with notable improvements in knowledge, attitudes, self-efficacy, and confidence observed after training [20]. The study clearly indicates that older children (aged 11‐17 years) outperform their younger counterparts (aged 7‐10 years) in key performance metrics, including compression depth and overall CPR quality.

Recent evidence [21] demonstrates that schoolchildren aged 10‐14 years can effectively perform chest compressions at rates consistent with AHA and European Resuscitation Council (ERC) guidelines, achieving the recommended 100‐120 compressions per minute in controlled training environments. However, younger students often face challenges in achieving the recommended chest compression depth due to physical factors such as height, weight, and strength [22]. Despite these challenges, consistent early CPR education fosters confidence and a positive attitude toward performing CPR, which is critical for effective bystander intervention [23]. The current body of evidence demonstrates that schoolchildren across various age groups face significant physiological barriers that render them largely incapable of performing effective chest compressions to a depth of 5‐6 cm according to established resuscitation guidelines. Multiple studies consistently reveal age-related performance deficits [15] showing that no child in the 9‐10 years age group could achieve the guideline-recommended compression depth, while only 19% of 11‐ to 12-year-olds and 45% of 13‐ to 14-years-olds met these standards. A study involving Korean elementary school students found that while younger children struggle with achieving the recommended chest compression depth, consistent training can improve their performance over time [20].

Alternative approaches, such as the foot chest compression method [24] compared to traditional hand placement, failed to demonstrate significant improvement in achieving correct compression depth (47% vs 45%; *P*=.76) among schoolchildren, indicating that the fundamental issue lies in force generation capacity rather than technique modification. In addition, age and physical attributes such as height and weight, which are indirectly related to BMI, play a crucial role in CPR performance [15]. Older children generally perform better, achieving the necessary compression depth and rate more frequently than younger children, regardless of sex and BMI [25].

### Gender and Socioeconomic Factor

Gender differences in CPR training reveal nuanced insights [21]. Finke et al [26] described a meta-analysis that gender does not independently affect CPR performance quality; age plays a more significant role, with older schoolchildren performing better regardless of gender. Female high school students often exhibit a higher willingness to perform CPR compared to their male counterparts, both before and after training [27]. This willingness is crucial for the overall effectiveness of CPR interventions in real-life scenarios. Despite this, knowledge and practical application of CPR among high school students, including females, remain moderate, with notable gaps in skills such as the breathing check method. The effectiveness of CPR training and student retention can vary by school type, whether public or private. In Heraklion, Greece, no significant differences in CPR knowledge and skill retention were found between students trained by health care professionals, schoolteachers, or peer students, suggesting that the instructor type, rather than the school environment, plays a crucial role [26]. Similarly, a quasi-experimental study in rural South India showed significant improvements in CPR knowledge among adolescents following a structured training program, indicating that well-designed training can be effective regardless of the school setting [27]. In Brazil, Fernandes et al [28] found that private school students performed better than public school students in assessments of prior knowledge, immediate learning, and delayed learning. This suggests differences in educational quality or resources between the 2 types of schools. Socioeconomic factors also play a significant role, as private school students often come from families with higher socioeconomic status, providing additional educational support and resources [29]. The Brazilian Education Development Index (IDEB) results consistently show superior performance by private school students compared to their public-school counterparts [30]. In addition, the COVID-19 pandemic highlighted disparities, with private schools better equipped to transition to remote learning, maintaining higher performance levels, and potentially impacting long-term learning [31].

### Technological Integration and Future Directions

Computer-based learning [32,33] tools can increase engagement among schoolchildren compared to traditional methods, such as internet-based programs [7] and simulations, making BLS training more appealing and memorable. Digital platforms [34] can provide standardized training, ensuring consistent quality and adherence to the latest guidelines, crucial in medical education and community CPR programs [35]. Computer-based BLS training allows students to learn independently, revisiting complex topics as needed, which can benefit younger learners.

Online platforms and applications can make BLS training more accessible, reaching a wider audience, including schools in remote or under-resourced areas, particularly in low-income resource countries with diminishing inequities. Advanced computer programs [36] can offer real-time feedback during BLS training, allowing students to correct techniques immediately and learn more effectively. Virtual and augmented reality can simulate real-life scenarios, providing immersive training experiences without the risks associated with practicing on live participants. Computer-based training tools [37] can collect student performance data, providing valuable insights for educators to tailor and improve the BLS curriculum.

Implementing technology-driven BLS programs requires significant resources, including hardware, software, and training for educators, which might be a barrier for some institutions. However, using freeware and open-source software can overcome these barriers. The rapidly evolving nature of medical guidelines necessitates regular updates to the training content, which can be resource intensive. While technology can enhance learning, balancing it with human interaction is crucial, primarily to address the emotional and psychological aspects of emergency response training.

### Impact of Training Methods

Recently published the 2024 cardiopulmonary resuscitation guidelines [38], recommending that the incorporation of gamified learning be considered as a component of resuscitation training for all BBLS and advanced life support (ALS) courses. This recommendation is made with a weak endorsement due to the current evidence being of very low quality. Nonetheless, gamification has the potential to enhance learner engagement and motivation [39], which may improve the overall effectiveness of these training programs. It can lead to a more informed and prepared generation capable of responding effectively to emergencies.

However, the success of this approach depends on addressing challenges related to resources and content management and maintaining a balance between technological and human elements in education. Continuous research and innovation in this field are essential to maximize the benefits of technology in BLS training for schoolchildren. Implementing novel pedagogical approaches, such as the flipped classroom model [40], has resulted in promising results when evaluating the influence of this method on BLS skills in elementary school children. The study revealed that these SG and student-centered strategies can improve learning outcomes. This method allows students to engage with the material at home and then apply what they have learned in a practical classroom setting, potentially leading to better skill acquisition and retention. Research conducted by Abelairas et al [41] evaluated the effectiveness of very short 4-month rolling refreshers was compared to that of annual retraining. The results indicated that increasing the frequency of training sessions while reducing their duration could enhance effectiveness compared to decreasing the frequency and increasing the duration of sessions. This strategy could facilitate the preservation of a high level of proficiency among pupils, guaranteeing their constant readiness to react in emergencies.

### Protocol Deviations

The study protocol was adaptively refined from its original approved form to better align with post-pandemic realities and opportunities for enhanced engagement. All modifications were formally documented and received IRB approval before implementation, ensuring the continued integrity and relevance of the research. In response to overwhelming interest from partner schools, the study population was expanded from two to twenty schools, enrolling 336 students. This adaptation allowed the research to extend its reach to a broader and more developmentally diverse cohort (ages 7‐17 years), maximizing the public health impact of the training.

Further refinements were made to the intervention and evaluation methodology to improve efficacy and feasibility. Leveraging additional resources, the training was optimized into a concise, 50-minute module comprising a video-based lesson and a gamified training component delivered via a serious game. Critically, the primary outcome measure was transitioned from a written test to direct, quantitative CPR performance metrics, including data from the serious game and QCPR scores. This essential change ensured equitable assessment for all participants, particularly younger children whose literacy was affected by pandemic-related educational disruptions. Consequently, the 6-month follow-up was deemed no longer applicable to the new primary endpoint. The study timeline was also adjusted to accommodate this expanded scope. All changes were guided by a primary commitment to participant welfare and scientific rigor.

### Limitations

This study has several methodological limitations that should be acknowledged. First, the single-center design conducted in one geographic region of Brazil may limit the generalizability of findings to other populations with different socioeconomic or educational contexts. The short-term evaluation period also precludes assessment of long-term skill retention, a critical factor for BLS training efficacy. While we controlled for key anthropometric variables, unmeasured confounders (cognitive development and prior gaming experience) may have influenced results. Finally, the study did not evaluate actual willingness to perform CPR in emergencies, a crucial behavioral outcome that may not correlate directly with manikin-based performance metrics. Future multicenter randomized trials with longer follow-up are needed to address these limitations.

### Recommendations

Future studies should investigate optimal training intervals through longitudinal designs assessing skill retention at 6‐12-month intervals. Multicenter randomized controlled trials comparing game-based training versus traditional methods are needed to establish causal efficacy, particularly in low-income countries. Research should also examine real-world translation by measuring actual bystander CPR rates among trained students. Finally, serious game developers should incorporate adaptive algorithms that adjust difficulty based on the user’s age and physical capabilities, while maintaining fidelity to evidence-based CPR guidelines. These evidence-based recommendations could significantly advance global implementation of the WHO’s Kids Save Lives initiative while addressing critical gaps in resuscitation education.

### Conclusion

This study demonstrates that a serious game-based intervention provides an effective and equitable method for delivering BLS training to schoolchildren. Multivariate analysis confirmed that while inherent physiological factors, specifically age, height, and grip strength, were independent predictors of higher CPR performance scores, the knowledge acquired from the game-based training was universal. Postintervention scenario performance improved significantly across all groups, with no observed disparities attributable to age or socioeconomic status.

## Abbreviations

AHA: American Heart Association
ALS: advanced life support
BLS: basic life support
CPR: cardiopulmonary resuscitation
ERC: European Resuscitation Council
IDEB: Brazilian Education Development Index
ILCOR: International Liaison Committee on Resuscitation
KSL: Kids Save Lives
OHCA: out-of-hospital cardiac arrest
QCPR: quality cardiopulmonary resuscitation
SG: serious game
STROBE: Strengthening the Reporting of Observational Studies in Epidemiology
WHO: World Health Organization
